# Multifunctional Nanomaterials for Energy Applications

**DOI:** 10.3390/nano12132170

**Published:** 2022-06-24

**Authors:** Simas Rackauskas, Federico Cesano, Mohammed Jasim Uddin

**Affiliations:** 1 Institute of Materials Science, Kaunas University of Technology, 44249 Kaunas, Lithuania; simas.rackauskas@ktu.lt; 2 Department of Chemistry, Turin University & INSTM-UdR Torino, 10125 Torino, Italy; 3 Photonics and Energy Research Laboratory-PERL, Department of Chemistry, The University of Texas Rio Grande Valley, Edinburg, TX 78539, USA; mohammed.uddin@utrgv.edu

In the last few decades, global energy requirements have grown exponentially, and increased demand is expected in the upcoming decades. Traditional energy resources have remarkably impacted energy production so far, but the use of renewable energy sources has constantly increased and is gradually substituting fossil fuels. Such non-renewable energy resources are limited in nature, and their use for energy purposes affects climate change. The new paradigm is materials for sustainable energy, and when materials are nanostructured, new key concepts are involved. Nanomaterials exhibit properties very different from their bulk counterparts due to their significant surface boundary and quantum confinement characteristics. Furthermore, the structure (or nanophase assembly) is also relevant for explaining various novel and interesting properties, notably when energy applications are taken into consideration. Remarkably, the aggregation and interface properties of nanostructures, even at lower dimensionality, are expected to boost energy applications.

Nanomaterials and nanotechnologies for energy have been more actively studied and used since the 2000s, as recognized by the number of scientific contributions that are growing exponentially (Figure 1a). As far as the geographical point of view is concerned, the subdivision of the contributions seems unbalanced when considering the continents: North/East/Central Asia (52%), Europe (23%), North/South America (18.3%), Africa (3.8%); Australia (2.5%). In more details, most contributions have been from China (c.a. 26.5%) and the United States (15.0 %), followed by India (9.8%), South Korea (4.3%), UK and Germany (2.9%), Australia, Japan, France (c.a. 2.5%), Italy (2%), and Spain (1.8%) (Figure 1b). This subdivision probably does not reflect the geographical distribution of investments, but it provides an overview of countries providing innovation in the near future in the fields of energy by nanomaterials. However, nanomaterials are not the only materials that have attracted the recent attention of the scientific community. For example, nanostructured materials and compounds are also frequently studied subjects.

As for the global market for energy nanomaterials, recent trends of segment indicate a significantly increasing of demand with a stable growth at a compound annual growth rate (CAGR) of 13.4% over the 2020–2027 period [1]. The global nanotechnology in energy industry was estimated to be at $140 million (2020), and it is estimated near $385 million by 2030 [2]. The geographical distributions of the market prospect a rising product application (especially in North America), the expanding mass production and price reduction of nanomaterials (mainly in Europe) together with the entry for new players due to the government financial support, increasing demand, along with a huge population (Asia-Pacific) [1,2].

From a thematic viewpoint, the field of “*energy materials*” is very wide the research requires a multidisciplinary approach with multifaceted activities from basic and fundamental scientific studies to more applicative works.

This Special Issue, comprising two reviews and fourteen research articles, highlights some recent improvements and perspectives in the field of energy nanomaterials, which can be considered to address our current improvements and future challenges. The number and variety of contributions reflect the remarkable interest in topics related to energy nanomaterials. Furthermore, the presence of contributions addressing environmental issues, such as the use of environmentally friendly and recyclable raw materials, indicates an increased interest in sustainability, which is likely to become in the near future an increasingly important issue.

From such a broad and multifaceted item, the reader can expect that the contributions will cover some of the most debated hot topics in the scientific community, including materials and technologies for: (i) light harvesting enabling efficiency improvements [3,4], (ii) triboelectric nanogenerators [5]; (iii) nuclear energy production [6]; (iv) energy (batteries, supercapacitors, and some components, including electrolytes, electrodes, catalysts) [7], and fuel (methanol, H_2_) storage [8]; CO_2_ capture; (v) thermal energy storage applications [9]; (vi) new wiring based on carbons for electrical applications and electronics [10,11]; and (vii) detectors, optoelectronic devices (i.e., laser technologies, spectral converters, LEDs and three-dimensional displays).

Oliveira et al. [12] reviewed the topic of colloidal lithography (CL) for photovoltaics (PVs). The authors overviewed some of the most promising methods for material micro/nano structuring methods in the field of photovoltaics, where colloidal lithography has been demonstrated to be among the best and preferred patterning techniques for implementation in industrial processes and promise to be a reliable alternative to conventional hard-patterning processes. In this regard, photonic-enhanced PVs have been shown to bypass many of the conventional shortcomings of current solar cell technologies, as follows: (i) reflection losses and (ii) decreased light absorption occurring in thin films. The authors remarked also on the opportunity to enable other attractive functionalities, such as improved transparent contacts and self-cleaning properties due to the high aspect ratio of photonic microstructures.

Song et al. [13] reported a non-iterative method to precisely extract five model parameters for a single diode model of solar cells. The authors’ method, overcoming the complexity and accuracy difficulties by employing a simplified calculation process, uses equation parts which are to be dynamically adapted, and thus the five parameters are calculated from the I-V curve. Interestingly, the authors reported that the proposed method is reliable (more than other methods) based on the root mean square error analysis. More interestingly, the authors simulated I-V and P-V characteristics by using the extracted parameters and compared them with the experimental fit of solar cells under different conditions.

Shaoo et al. [14] reported the fabrication of a flexible multilayer graphene triboelectric nanogenerator (TENG) for use as an energy harvester for next-generation flexible electronics. The device was layer-by-layer assembled by introducing a charge trapping layer (CTL) made of Al_2_O_3_, which was placed between the conducting electrode and the positive triboelectric layer. The authors reported a 30-fold increase in output power for three layers of graphene TENG (3L-Gr-TENG) with CTL compared to 3L-Gr-TENG without CTL device counterpart. Interestingly, the developed device also showed continuous operations for more than 2000 cycles with remarkable stability. Surprisingly, the same device was capable of powering 20 green LEDs and sufficient to power an electronic timer with rectifier circuits. The authors associated the greatly improved performance with the synergistic effects occurring between the graphene layers and the Al_2_O_3_ dielectric layer.

Durairaj et al. [15] reviewed the cellulose nanocrystals (CNCs) field for the application as supercapacitors (SCs), from CNC synthesis/surface function to development of conductive CNCs. The authors also remarked and summarized recent perspectives and problems to be addressed including: (i) the importance of fabricating CNCs of millimeter thickness and with a hierarchical porous microstructure; (ii) control/optimization of CNC surface for both hydrophobic and hydrophilic matrices; (iii) bio-precursors allowing the incorporation of biological molecules (i.e., virus, etc.) for making a better impact on their superior properties; (iv) the need to obtain metal-free porous and heteroatom-doped CNC using a low cost, and sustainable approach from abundant biomass; and (v) the need to improve the performance of SCs, energy, and power densities of SCs.

Bakardjieva et al. [16] synthesized layered ternary Ti_2_SnC carbides. Among the adopted synthesis methods, an unconventional low-energy ion facility (LEIF) based on Ar+ ion beam sputtering of Ti, Sn, and C targets, was adopted by the authors. The contribution provided insights into the understanding of Sn atom segregation at the surface, highlighting the role played by aberration-corrected STEM techniques, such as high-angle annular dark-field detector (HAADF) analysis combined with simulations of SAED patterns to track atomic paths clarifying the properties of Sn atoms at the proximity of irradiation-induced nanoscale defects and the existence of oxidized species formed during the preparation process.

Veelken et al. [17] investigated the local ionic conductivity in a hybrid electrolyte interface between ceramic particles and polymers in hybrid electrolytes (polyethylene oxide with Li bis(trifluoromethanesulfonyl)imide: PEO_6_–LiTFSI; and Li_6.5_La_3_Zr_1.5_Ta_0.5_O_12_: LLZO:Ta) by electrochemical strain microscopy (ESM). Interestingly, the results presented by the authors described significant insights to attain an advanced understanding of ionic transport mechanisms in the interior of hybrid electrolytes, and provided two strategies for the hybrid solid-state electrolyte improvements. Firstly, the covering of the particles can help to decrease the interfacial resistance. Secondly, a structure consisting of a continuous percolation path along the ceramic particles may take advantage of the high lithium content at the interfacial regions with the consequent overall ionic conductivity increasing.

Cui et al. [18] reported the fabrication of a graphene-polydopamine electrode (PDA@3DVAG) composite with 3D vertical-oriented macropores by unidirectional freezing and subsequent self-polymerization approach. The authors tested the composite as the positive electrode of zinc-ion hybrid supercapacitors (ZHSCs) and reported excellent electrochemical performances compared with the conventional electrolyte. In this regard, the authors reported that the vertically oriented composite electrode showed better properties compared to performance (48.92% at a current density of 3 A g^−1^), wider voltage window (±0.8 V voltage drop), better cycle performance with specific capacitance from 96.7 to 59.8 F g^−1^, and higher energy density (46.14 Wh kg^−1^).

González-Gil et al. [19] prepared a new nanocellulose-based gel polymer electrolyte (GPE) to be used in supercapacitors. The authors synthesized the GPE from a mixture of an ionic liquid (1-ethyl-3-methylimidazolium dimethyl phosphate) with carboxymethylated cellulose nanofibers at different weight ratios. The addition of nanocellulose-based fibers helped to improve the ionogel properties, including ionic conductivity in the 0.32–0.94 mS cm^−2^ interval and to become easily printable on the electrode surface. Interestingly, the authors reported that the new GPE-based supercapacitor cell showed good electrochemical performances with high specific capacitance (160 F g^−1^) excellent energy density and good power density (46.14 Wh kg^−1^ at the power density of 393.75 W kg^−1^ and 19.29 Wh kg^−1^ at 2183 W kg^−1^, respectively). More interestingly, the energy density exceeds that of conventional electrochemical capacitors and of some large-size batteries.

Askari et al. [20] reported the one-step preparation by the hydrothermal method of Mn_3_O_4_-CeO_2_ mixed metal oxides and reduced graphene oxide (rGO) hybrid catalysts to be used in the methanol oxidation reaction (MOR) process. Interestingly, the authors remarked the occurrence of a synergetic effect occurring between rGO and Mn_3_O_4_-CeO_2_. In this domain, Mn_3_O_4_-CeO_2_-rGO material showed an oxidation current density of 17.7 mA/cm^2^ in overpotential of 0.51 V and 91% stability after 500 consecutive runs of cyclic voltammetry (CV). Furthermore, the optimal concentrations of methanol for Mn_3_O_4_-CeO_2_ and Mn_3_O_4_-CeO_2_-rGO catalysts were determined in CV experiments to be 0.6 and 0.8 M, respectively.

Wan et al. [21] synthesized a series of amine-functionalized highly stable Ti-based MOFs (MIP-207) by the mixed linkers method from 1,3,5-benzenetricarboxylic acid (H3BTC) and 5-aminoisophthalic acid (C_8_H_7_NO_4_) with different weight fractions. Interestingly, the introduction of amino groups demonstrated remarkable CO_2_ uptake performance (up to 3.96 and 2.91 mmol g^−1^), which are 20.7% and 43.3% higher than those of unmodified MIP-207 at 0 and 25 °C, respectively. The CO_2_ uptake is attributed by the authors to the introduction of -NH_2_ into the framework of MIP-207, leading to the increase of specific surface area and more Lewis basic adsorption sites, thereby enhancing the CO_2_ working capacity and CO_2_/N_2_ selectivity properties.

Bu et al. [22] synthesized Co nanoparticles supported on bagasse-derived porous carbon catalysts for catalyzed hydrolytic dehydrogenation reaction of NaBH_4_. One of the catalysts prepared by the authors exhibited a remarkable hydrogen generation activity with an optimal H_2_ production rate of 11,086.4 mL_H2_∙min^−1^∙g_Co_^−1^ and low activation energy (31.25 kJ mol^−1^). Density functional theory (DFT) results indicated that the metal nanoparticles-supported on porous carbon catalyst structure was advantageous for the dissociation of [BH_4_]^−^, which remarkably enhanced the hydrolysis efficiency of NaBH_4_. Interestingly, the catalyst presents excellent durability, retaining 72.0% of the initial catalyst activity after several cycling tests.

Mahroug et al. [23] reported a selection of the most promising oxide-based supporting materials for the Li_4_(OH)_3_Br peritectic compound to be used in thermal energy storage (TES) applications at ca. 300 °C. The authors investigated micro/nanoparticles of MgO, Fe_2_O_3_, CuO, SiO_2_ and Al_2_O_3_ as candidates for supporting materials. Among all oxides, based on: (i) chemical compatibility of the supporting material with molten Li_4_(OH)_3_Br; (ii) anti-leakage effectiveness and maximum salt loading; and (iii) thermal and microstructural properties and stability of Li_4_(OH)_3_B, MgO nanoparticles were found the most promising oxide also due to the fact that all of the other oxides studied showed more or less pronounced upon heating and upon cycling conditions.

Orbaek White et al. [24] reported the growth of multi-walled car-bon nanotubes (MWCNTs) by the liquid injection chemical vapor deposition (LI-CVD) method at 780 °C from toluene-loaded polystyrene (PS) with different amounts of PS in the presence of ferrocene used as a catalyst. Then, acid-washed Bucky papers were produced, and their DC electrical properties were found to be in the range of 2.2–4.4 Ohm, with no direct correlation with PS loading. In addition, MWCNTs were used by the authors to fabricate MWCNT-based ethernet cables, consisting of tightly packed MWCNT powders. These 2-cm long carbon wires were then tested in server-to-client data transfer operations. Interestingly, the authors measured data transfer rates up to about 99 Mbps for the MWCNT-based wires. More interestingly, the life cycle assessment (LCA) of MWCNT-based wires was compared to that of copper ones for a use-case scenario in a Boeing 747-400 airliner over its lifetime. Due to their lightweight properties, MWCNT wires were found to reduce the CO_2_ footprint by 21 kTonnes (kTe) over the overall life of the aircraft.

Louw et al. [25] modelled a new proposed Fe^+++^ ion sensor based on Eu-MOF crystals placed on a polymer surface. In their paper, the diffusion properties of ferric ions through a solution and a polymer layer and the interaction with a MOF crystal located at the interface between the solution and the polymer were investigated. The authors adopted a 2D diffusion model to predict the progress of Fe^+++^ through the solution and the polymer, and the association of Fe^+++^ with a MOF crystal at the interface. In the paper, a facile 1D model was reported to find the most appropriate values for the dimensionless parameters required to optimize the time for a MOF crystal to reach a steady state. Interestingly, a large non-dimensional diffusion coefficient was obtained and a small effective flux association reducing the time to reach a steady state was predicted by the authors’ model.

Pawlin et al. [26] reported the synthesis of multicolor light-emitting nanomaterials based on Tb^3+^ and Eu^3+^ rare earth co-doped oxyfluoride glass-ceramics containing BaF_2_ nanocrystals. Excitation and emission spectroscopy along with decay analysis from the ^5^D_4_ level of Tb^3+^ was performed and the authors observed that co-doping with Eu^3+^ caused the decrease in decay times of the ^5^D_4_ state from 1.11 ms to 0.88 ms and from 6.56 ms to 4.06 ms for xerogels and glass-ceramics, respectively. Hence, based on lifetime values, the Tb^3+^/Eu^3+^ energy transfer (ET) efficiencies were estimated to be ca. 21% for xerogels and 38% for nano-glass-ceramics. The authors explained the increase in energy transfer efficiency by shortening the separation between interacting Tb^3+^ and Eu^3+^ cations embedded into the BaF_2_ nanocrystal lattice.

We hope that contributions collected in this Special Issue may benefit researchers and experts in various fields for growing their knowledge in the fields of energy nanomaterials, thus stimulating new relevant studies. We also convey our truthful appreciation to the editorial staff, authors, and referees for their constant and rapid support, beneficial contributions, and appropriate comments.

## Figures and Tables

**Figure 1:**
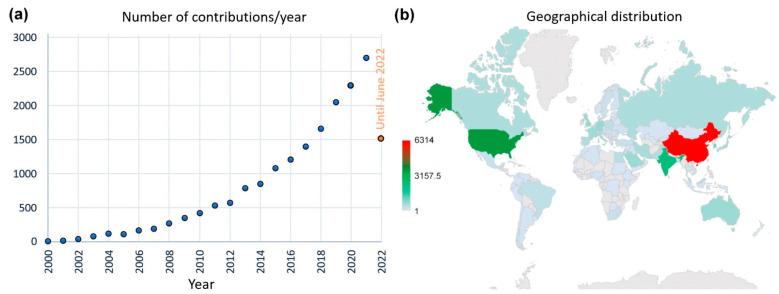
Scientific contributions dedicated to energy nanomaterials: (**a**) documents published since 2000 and (**b**) their geographical distribution. Keywords: “energy” AND “nanomaterials” within: Article title, Abstract, Keywords (Source: Scopus; May 2022).

## Abbreviations

CL: colloidal lithography; CNCs: cellulose nanocrystals; CTL: charge trapping layer; CV: cyclic voltammetry; DC: direct current; DFT: density functional theory; ESM: electrochemical strain microscopy; ESR: equivalent series resistance; GPE: gel polymer electrolyte; HAADF: high-angle annular dark-field detector; LCA: lifecycle assessment; LEIF: low-energy ion facility; LI-CVD: liquid injection chemical vapour deposition; LLZO:Ta: Li6.5La3Zr1.5Ta0.5O12; MIP-207: Ti-based metal-organic framework, MOFs: metal-organic frameworks; MOR: methanol oxidation reaction; MWCNTs: multiwalled carbon nanotubes; PCM: phase change material; PEO_6_–LiTFSI: polyethylene oxide with Li bis(trifluoromethanesulfonyl)imide; PV: photovoltaic; rGO: reduced graphene oxide; SAED: selected area electron diffraction; SCs: supercapacitors; TENGs: triboelectric nanogenerators; TES: thermal energy storage; ZHSCs: zinc-ion hybrid supercapacitors.
